# Supplementary material for: Hybrid Models and Biological Model Reduction with PyDSTool
Source: PLoS Comput Biol. 2012 Aug 9;8(8):e1002628. doi: 10.1371/journal.pcbi.1002628 (PMC3415397; doi:10.1371/journal.pcbi.1002628)
Supplement: Text S4 — Complete source code for the PyDSTool package (version 0.88.120504). Includes API documentation and help files linking to web pages. This file is identical to the current public release on Sourceforge.net. (ZIP) [file pcbi.1002628.s004.zip › PyDSTool/html/PyDSTool.MProject.feature_leaf-class.html]

xml version="1.0" encoding="ascii"?


PyDSTool.MProject.feature\_leaf


| Home | Trees | Indices | Help | PyDSTool |
| --- | --- | --- | --- | --- |

Package PyDSTool ::
Module MProject ::
Class feature\_leaf

[hide private]

[frames] | no frames]

# Class feature\_leaf

source code

```
object --+    
         |    
   feature --+
             |
            feature_leaf
```

Known Subclasses:
:   - qt\_feature\_leaf
    - , ql\_feature\_leaf

---

Abstract super-class for feature leaf nodes.


|  |  |
| --- | --- |
| Instance Methods | [hide private] |

|  |  |
| --- | --- |
| set\_ref\_traj(self, ref\_traj)  May or may not be used by the feature. | source code |

|  |  |
| --- | --- |
| \_residual\_info(self, feats, sizes)  Update feats and sizes lists in place with metric info, if any. | source code |

|  |  |
| --- | --- |
| \_\_str\_\_(self)  str(x) | source code |

|  |  |
| --- | --- |
| \_\_repr\_\_(self)  str(x) | source code |

**Inherited from `feature`**:
`__call__`,
`__eq__`,
`__hash__`,
`__init__`,
`__ne__`,
`evaluate`,
`finish`,
`info`,
`postprocess_ref_traj`,
`prepare`,
`reset_metric`,
`validate`

**Inherited from `feature`** (private):
`_find_idx`

**Inherited from `object`**:
`__delattr__`,
`__getattribute__`,
`__new__`,
`__reduce__`,
`__reduce_ex__`,
`__setattr__`


|  |  |
| --- | --- |
| Properties | [hide private] |

**Inherited from `object`**:
`__class__`


|  |  |
| --- | --- |
| Method Details | [hide private] |

|  |  |
| --- | --- |
| set\_ref\_traj(self, ref\_traj) | source code |

May or may not be used by the feature. If not used, it will be
ignored.

Overrides: feature.set\_ref\_traj: *(inherited documentation)*

|  |  |
| --- | --- |
| \_\_str\_\_(self)  *(Informal representation operator)* | source code |

str(x)

Overrides: object.\_\_str\_\_: *(inherited documentation)*

|  |  |
| --- | --- |
| \_\_repr\_\_(self)  *(Representation operator)* | source code |

str(x)

Overrides: object.\_\_repr\_\_: *(inherited documentation)*

  


| Home | Trees | Indices | Help | PyDSTool |
| --- | --- | --- | --- | --- |

|  |  |
| --- | --- |
| Generated by Epydoc 3.0.1 on Fri May 4 15:24:06 2012 | http://epydoc.sourceforge.net |
